# O-GlcNAcylation Is Essential for Autophagy in Cardiomyocytes

**DOI:** 10.1155/2020/5602396

**Published:** 2020-08-11

**Authors:** Houzhi Yu, Li Wen, Yongxin Mu

**Affiliations:** ^1^Department of Cardiology, Shandong Provincial Hospital Affiliated to Shandong First Medical University, Jinan, China; ^2^Department of Medicine-Cardiology, University of California San Diego, 9500 Gilman Drive, Mail Code 0613-C, La Jolla California 92093-0613, USA; ^3^Department of Neurology, Shandong Provincial Hospital Affiliated to Shandong First Medical University, Jinan, China

## Abstract

Since both O-GlcNAcylation and autophagy sense intracellular nutrient level, the alteration of those two pathways plays substantial roles in the progression of heart failure. Hence, determining the relationship between O-GlcNAcylation and autophagy is imperative to understand, prevent, and treat heart failure. However, the mechanism on how O-GlcNAcylation regulates autophagy in the heart is poorly investigated. In this study, we demonstrated that O-GlcNAcylation is required for autophagy in cardiomyocytes by utilizing an O-linked *β*-N-acetylglucosamine transferase (OGT) cardiomyocyte-specific knockout mouse model for the first time. We also identified that OGT might regulate the initiation of autophagy in cardiomyocytes through promoting the activity of ULK1 by O-GlcNAcylation. In conclusion, our findings provide new insights into the molecular mechanisms underlying heart dysfunction and benefit the development of treatments for heart failure.

## 1. Introduction

O-GlcNAcylation is an important posttranslation modification of proteins by the addition of O-linked *β*-N-acetylglucosamine (O-GlcNAc) moieties at serine or threonine residues. Similar to protein phosphorylation, which also occurs on serine or threonine residues, O-GlcNAcylation is dynamic. In contrast to phosphorylation, which is catalyzed and removed by hundreds of kinases and phosphatases with relative substrate specificities, O-GlcNAcylation is catalyzed and removed by a single pair of enzymes, O-GlcNAc transferase (OGT) and O-GlcNAcase (OGA), respectively [1, 2]. Increased O-GlcNAcylation in a hypertrophic or failing heart has been reported from human patients and animal models [3, 4]. Additionally, previous studies using both *in vitro* and *in vivo* models have suggested that increased O-GlcNAcylation plays a cardioprotective role in acute cardiac dysfunctions, yet it may have deleterious effects on cardiac function in chronic conditions [3]. Genetic knockout of OGT at embryonic stage in mice led to congenital heart diseases, resulting in partial postnatal lethality [5] and cardiac hypertrophy among surviving mice [6]. The acute loss of OGT in cardiomyocytes also exacerbated heart failure induced by myocardium ischemia [7].

Autophagy is a conserved mechanism for the degradation of intracellular elements and plays an essential role in protein homeostasis and the quality control of subcellular organelles [8–12]. Although sometimes autophagy can induce cell death with unique morphological changes, in most cases, autophagy plays protective or adaptive roles and prevents cell death [12, 13]. Autophagy is essential in maintaining cardiac structure and function at both baseline by degrading misfolded proteins and damaged organelles and during stress by limiting the cardiac damage in different pathological conditions such as ischemia, starvation, and hemodynamic overload [14, 15]. A decreased level of autophagy was proven to contribute to the progression of heart failure and aging [16, 17]. Hence, autophagy plays an important role in mediating cardiac homeostasis and adaption to aging, stress, and myocardial injury.

Recently, the relationship between O-GlcNAcylation and autophagy is gaining more interests, and studies have shown that O-GlcNAcylation indeed regulates autophagy [18, 19]. Some reports have shown that O-GlcNAcylation negatively regulates autophagy in the heart. Noteworthy, those studies have used Streptozotocin (STZ) to increase O-GlcNAcylation *in vivo* [20, 21] by damaging pancreatic *β* cells [22]. However, damaged *β* cells and STZ's well-known side effects on other organs [22] may compromise the relationship between O-GlcNAcylation and autophagy specifically in the heart or cardiomyocytes.

Here, we used the Cre-Loxp system to specifically knock out OGT and abolish O-GlcNAcylation in cardiomyocytes. We found that the loss of O-GlcNAcylation in cardiomyocytes attenuated autophagy, especially under fasting condition. Also, data from isolated neonatal cardiomyocytes showed that the loss of OGT affected the early stage of autophagy. Moreover, we identified that Unc-51-Like Autophagy Activating Kinase 1 (ULK1), an essential kinase for initiating autophagy flux, was O-GlcNAcylated in cardiomyocytes, and the level of O-GlcNAcylation of ULK1 was diminished in OGT knockout cardiomyocytes. Taken together, our data demonstrated that O-GlcNAcylation is essential for the initiation of autophagy in cardiomyocytes. Our findings provide novel insights on the regulation of autophagy in heart diseases.

## 2. Materials and Methods

### 2.1. Animal Models

*Ogt* ^flox/flox(f/f)^ mouse was purchased from Jackson Lab (Stock No: 004860 | OGTF). Floxed female mice were crossed with *α-Mhc-MerCreMer* transgenic mice [23] to create *Ogt*^f/y^; *α*-*Mhc-MerCreMer*-inducible KO (icKO) mice. All mice were of a mixed 129/SvJ and C57BL/6J background. Genotypes of the mice were confirmed by polymerase chain reaction (PCR) analysis using tail genomic DNA and *Ogt* primers (forward: 5′-CATCTCTCCAGCCCCACAAACTG-3′, reverse: 5′-GACGAAGCAGGAGGGGAGAGCAC-3′) and *Cre* primers (forward: 5′-GTTCGCAAGAACCTGATGGACA-3′; reverse: 5′-CTAGAGCCTGTTTTGCACGTTC-3′). All animal procedures were performed in accordance with the National Institutes of Health Guide for the Care and Use of Laboratory Animals and approved by the Institutional Animal Care and Use Committee of the University of California, San Diego, with an approved protocol# S01049.

### 2.2. Tamoxifen Induction

4-Hydroxytamoxifen was dissolved in sesame oil at a concentration of 10 mg/mL. Adult (2-month-old) *Ogt*^f/y^ and *Ogt*^f/y^; *α-Mhc-MerCreMer* mice were treated with 4-hydroxytamoxifen by intraperitoneal injection once daily for 5 days with a dosage of 30 mg/kg body weight. Ten days after the last dose of tamoxifen, mice were either given unlimited food or fasted for 12 hours, followed by heart collection for western blot analysis.

### 2.3. Adenoviral Vectors, Reagents, and Antibodies

Adenoviruses expressing Cre and lacZ (Ad-Cre and Ad-lacZ) were obtained from the UCSD Viral Vector Core. Adenovirus expressing mRFP-GFP-LC3 (Ad-tf-LC3) was provided as a gift from Dr. Junichi Sadoshima. 4-Hydroxytamoxifen (H7904), Thiamet-G (TMG, SML0244), and Bafilomycin A1 (SML1661) were purchased from Sigma-Aldrich. Antibodies used in this study included RL2 (MA1-072, Thermo Fisher Scientific), OGT (61355, Active Motif), ULK1 (4773, Cell Signaling), pATG16L1 (ab195242, Abcam), LC3B (2775, Cell Signaling), SQSTM1 (GP61-C, Progen), ubiquitin (sc8017, Santa Cruz), and GAPDH (sc365062, Santa Cruz).

### 2.4. Protein Isolation and Western Blot Analysis

Total protein extracts were prepared by suspending ground heart tissue or isolated cardiomyocytes in a urea lysis buffer (8 M urea, 2 M thiourea, 3% SDS, 75 mM DTT, 0.03% bromophenol blue, 0.05 M Tris-HCl, pH 6.8). Protein lysates were separated on 4% to 12% SDS-PAGE gels (Thermo Fisher Scientific) and transferred at 4°C overnight onto nitrocellulose membranes (Bio-Rad). After blocking for 1 hour in TBS containing 0.1% Tween-20 (TBST) and 5% dry milk, the membranes were incubated at 4°C overnight with the indicated primary antibodies in a blocking buffer. Blots were washed and incubated with the appropriate HRP-conjugated secondary antibodies (1 : 5000) for 1 hour at room temperature. Immunoreactive protein bands were visualized using an ECL reagent (Thermo Fisher Scientific).

### 2.5. Immunoprecipitation

After the transduction with Ad-LacZ or Ad-Cre viruses for 48 hours, neonatal cardiomyocytes were lysed in a RIPA lysis buffer (50 mM Tris, 10 mM EDTA, 150 mM NaCl, 0.25% deoxycholic acid, 0.1% SDS, 2% NP-40 substitute, and 0.01% sodium azide). Cell lysates were rotated in the RIPA buffer with 10 *μ*L of RL2 or ULK1 antibody at 4°C overnight. Normal IgG (Santa Cruz) was used as a negative control. Next, 25 *μ*L of PBS-washed protein G beads (Thermo Scientific) were resuspended and incubated in the lysate-antibody complexes for 2 hours at 4°C. After washing 3 times with the RIPA lysis buffer, beads were incubated in 4 × LDS buffer (BioRad) at 70°C for 10 minutes, and the supernatants were collected. The immunoprecipitates and input lysate were gel electrophoresed and immunoblotted with the antibodies against O-GlcNAc (RL2) and ULK1.

### 2.6. Neonatal Mouse Cardiomyocyte Isolation and Treatments

Neonatal mouse cardiomyocytes were prepared as previously described [24] and maintained in Dulbecco's modified Eagle's medium (DMEM) supplemented with 10% horse serum, 5% fetal bovine serum (FBS), 100 U/mL penicillin, and 100 *μ*g/mL streptomycin for 24 hours before adenovirus transduction or other treatments. After being transduced with adenoviruses at MOI of 50, cardiomyocytes were cultured for additional 36 or 48 hours. Wild-type neonatal cardiomyocytes were treated with TMG (25 *μ*M) for 48 hours before other treatments. Right before collection, cardiomyocytes were treated with Bafilomycin A1 (100 nM) for 4 hours or subjected to starvation with serum-free medium for 18 hours.

### 2.7. Fluorescent Microscopy

48 hours after the transduction with Ad-tf-LC3 along with Ad-LacZ or Ad-Cre, cardiomyocytes were fixed with 4% paraformaldehyde for 5 min before confocal imaging. For immunostaining of pATG16L1, Ad-LacZ or Ad-Cre, transduced neonatal cardiomyocytes were fixed with 4% paraformaldehyde for 5 min and blocked in the blocking buffer (PBS with 1% BSA, 5% donkey serum, and 0.2% Triton 100) for 2 hours. They were then incubated with pATG16L1 antibody (1 : 200 in blocking buffer) overnight followed by a secondary antibody (1 : 300 in blocking buffer) incubation for 2 hours and confocal imaging.

### 2.8. Statistics

Data were presented as the mean ± SEM unless indicated otherwise. Statistical analysis was performed using GraphPad Prism 6.0 (GraphPad Software), with a 2-tailed Student's *t* test. *P* values of less than 0.05 were considered statistically significant.

## 3. Results

### 3.1. Deletion of Cardiomyocyte OGT Leads to the Attenuation of Autophagy in the Mouse Heart

**Figure 1:**
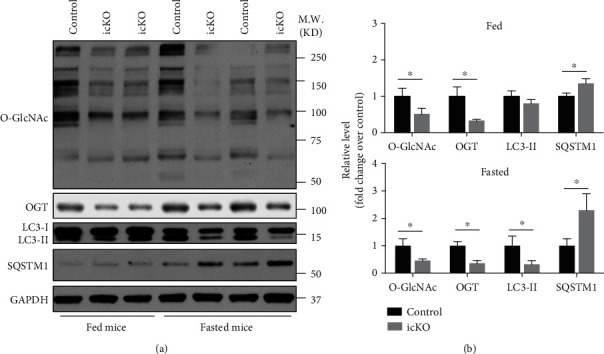
Acute loss of OGT attenuates autophagy in cardiomyocyte. (a) Western blot analysis of O-GlcNAc (RL2), OGT, LC3, and SQSTM1 using whole heart lysates from control and icKO mice with either food (fed) or water only (fasted). (b) Statistical analyses of the western blot results, *n* = 4 for each group. ^∗^Significantly different; M.W.: molecular weight.

To explore the possible effects of O-GlcNAcylation on autophagy in the heart, we crossed *Ogt*^f/f^ females with inducible *α-MHC-MerCreMer* male mice to generate *Ogt*^f/y^; *α-MHC-MerCreMer* mice, which were injected with tamoxifen for 5 days to generate OGT cardiac knockouts (hereafter icKO). Meanwhile, age-matched *Ogt*^f/y^ mice injected with the same doses of tamoxifen were used as controls. Ten days after the last dose of tamoxifen, when the icKO mice did not show heart dysfunction [6], western blot with RL2 and OGT antibodies showed that global O-GlcNAcylation and OGT dramatically decreased in icKO hearts. Under fed condition, the LC3-II level in the heart was not statistically different between control and icKO mice, although LC3-II in icKO mice was decreased (Figure 1). In contrast, the SQSTM1 level in icKO mice increased slightly but significantly (Figure 1). The most potent known physiological inducer of autophagy is starvation, and fasting has been widely used to investigate autophagy in mouse models [25]. Thus, we next investigated whether O-GlcNAcylation affected cardiac autophagy when mice were subjected to fasting. Surprisingly, the LC3-II level was significantly decreased in icKO mice when they were subjected to fasting. The SQSTM1 level in icKO mouse heart was further elevated accordingly (Figure 1). These data suggested that O-GlcNAcylation in cardiomyocytes is indispensable for autophagy under fasting condition, while it has only a mild influence on autophagy at the basal level.

### 3.2. Deletion of OGT in Isolated Neonatal Cardiomyocytes Attenuates Autophagy

**Figure 2:**
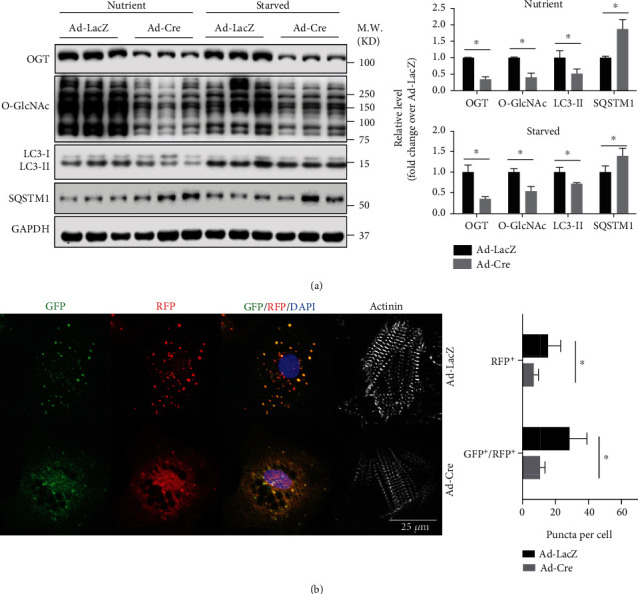
Deletion of OGT in isolated neonatal cardiomyocyte attenuates autophagy. (a) Left panel, western blot analysis for autophagy of Ad-LacZ or Ad-Cre-infected *Ogt*^f/f^ and/or *Ogt*^f/y^ neonatal cardiomyocytes with either full medium (nutrient) or starving medium (starvation), using antibodies against OGT, O-GlcNAc, LC3, and SQSTM1. GAPDH was detected as a loading control. Right panel, quantification of western blot results in the left panel, *n* = 3 for each group. ^∗^Significantly different; M.W.: molecular weight. (b) Left panel, representative fluorescent images of adenovirus RFP-GFP-LC3-infected control (Ad-LacZ treated) and knockout (Ad-Cre treated) *Ogt*^f/f^ and/or *Ogt*^f/y^ neonatal cardiomyocytes. Quantitative analyses of LC3 puncta per cell were shown in the right panel. 50 cells were analyzed for each group. ^∗^Significantly different.

To investigate whether OGT regulated autophagy in cardiomyocytes in a cell autonomous manner, we isolated neonatal cardiomyocytes from newborn pups from *Ogt*^f/f^ female and *Ogt*^f/y^ crossings. Those isolated neonatal cardiomyocytes were transduced with adenovirus expressing Cre recombinase (Ad-Cre) to delete OGT. Cells transduced with adenovirus expressing LacZ (Ad-LacZ) were used as control. Western blot showed that the levels of O-GlcNAcylation and OGT were dramatically decreased in Ad-Cre-treated cells (Figure 2(a)). LC3-II was significantly downregulated in knockout cells, regardless whether the cells were cultured in complete medium (nutrient) or subjected to starvation (starved) as described in Materials and Methods. Also consistent with the previous *in vivo* result (Figure 1), SQSTM1 was increased in knockout cells under both nutrient and starved conditions (Figure 2(a)). To further confirm the attenuation of autophagy in OGT knockout cardiomyocytes, the cardiomyocytes were transduced with adenovirus expressing tandem fluorescent mRFP-GFP-LC3 (Ad-tf-LC3). Ad-tf-LC3 allows the detailed monitoring of autophagy flux, because LC3 puncta labeled with GFP and mRFP represent autophagosomes, whereas those labeled with mRFP alone represent autolysosomes [26]. Indeed, under nutrient condition, the numbers of both mRFP-labeled autolysosomes and yellow autophagosomes were decreased in OGT knockout cardiomyocytes (Figure 2(b)). Collectively, our data suggested that O-GlcNAcylation is required for autophagy in mouse cardiomyocytes.

### 3.3. OGT Regulates the Early Stages of Autophagy in Cardiomyocytes

**Figure 3:**
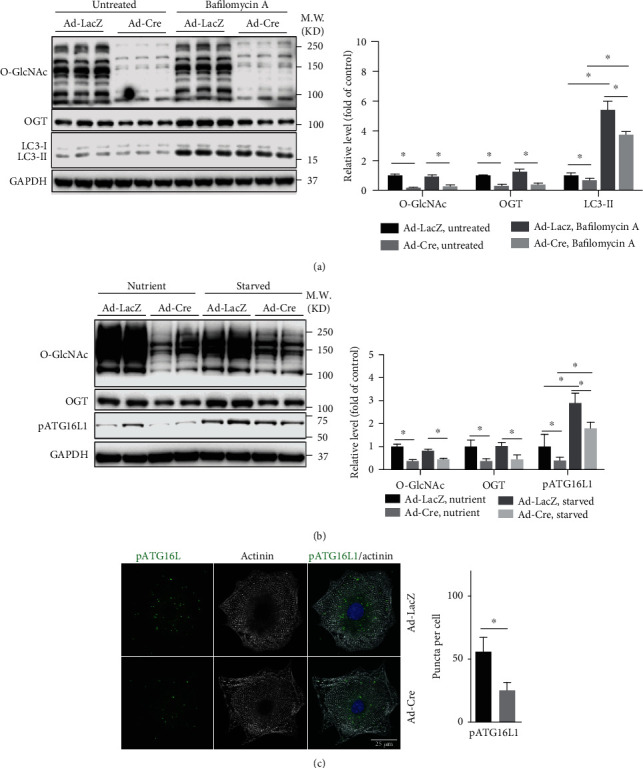
OGT regulates the early stage of autophagy in cardiomyocytes. (a) Left panel, western blot analysis for autophagy of untreated or Bafilomycin A-treated Ad-LacZ or Ad-Cre-infected *Ogt*^f/f^ neonatal cardiomyocytes, using antibody against O-GlcNAc, OGT, and LC3. GAPDH was detected as loading control. Right panel, quantification of western blot results in left panel, *n* = 3 for each group. ^∗^Significantly different; M.W.: molecular weight. (b) Left panel, western blot analysis for autophagy of Ad-LacZ- or Ad-Cre-infected *Ogt*^f/f^ and/or *Ogt*^f/y^ neonatal cardiomyocytes with either full medium (nutrient) or starving medium (starved), using antibody against pATG16L. GAPDH was detected as a loading control. Right panel, quantification of western blot results in the left panel, *n* = 4 for each group. ^∗^Significantly different; M.W.: molecular weight. (c) Left panel, representative immunomicroscopic images of pATG16L puncta (green) in Ad-LacZ- and Ad-Cre-infected *Ogt*^f/f^ and/or *Ogt*^f/y^ neonatal cardiomyocytes, costained with alpha-actinin (gray) and DAPI (blue). Right panel, quantification of pATG16L-positive puncta per cell, *n* = 50 cells each group. ^∗^Significantly different.

To further investigate how OGT regulates autophagy in cardiomyocytes, we treated the control and OGT deleted neonatal cardiomyocytes with or without Bafilomycin A (BafA), a commonly used inhibitor of autophagy through preventing autophagosome-lysosome fusion and acidification of lysosome [27]. Clearly, western blot showed that BafA treatment induced an accumulation of LC3-II in control cardiomyocytes but to a much lower extent in OGT deleted cardiomyocytes (Figure 3(a)). Those data indicated that the loss of OGT in cardiomyocytes attenuated the early stages of autophagy flux. A recent report has shown that the level of phosphorylated ATG16L1 (pATG16L1) correlates with the amount of newly formed autophagosome, and it has suggested that the pATG16L1 level could determine the rate of autophagy [28]. Hence, we took advantage of the newly generated antibody that recognizes pATG16L1 to confirm our finding. Consistently, western blot showed that the level of pATG16L1 was decreased in OGT deleted cardiomyocytes, under both nutrient and starved conditions (Figure 3(b)). Immunostaining also showed that the number of pATG16L1-positive puncta in OGT knockout neonatal cardiomyocytes under nutrient condition was decreased (Figure 3(c)). These data suggested that the loss of OGT in cardiomyocytes most likely affects autophagy induction rather than the late stages of autophagy.

### 3.4. Elevated O-GlcNAcylation in Neonatal Mouse Cardiomyocytes Promotes Autophagy

**Figure 4:**
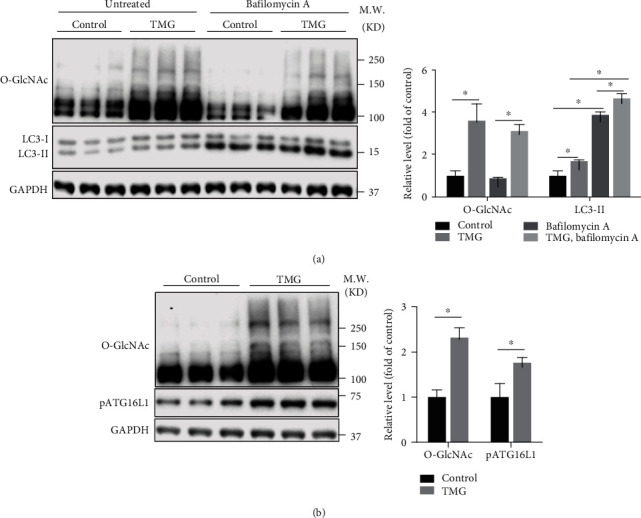
Enhanced O-GlcNAcylation stimulates autophagy in cardiomyocytes. (a) Left panel, western blot analysis for autophagy of wild-type neonatal cardiomyocytes with different treatments as indicated, using antibody against O-GlcNAc and LC3. GAPDH was detected as a loading control. Right panel, quantification of western blot results in the left panel, *n* = 3 for each group. ^∗^Significantly different; M.W.: molecular weight. (b) Left panel, western blot analysis of control or TMG-treated wild-type neonatal cardiomyocytes, using antibodies against O-GlcNAc and pATG16L. GAPDH was detected as a loading control. Right panel, quantification of western blot results in the left panel, *n* = 3 for each group. ^∗^Significantly different; M.W.: molecular weight.

To check whether elevated O-GlcNAcylation in cardiomyocytes has the opposite effect on autophagy, we treated wild-type neonatal mouse cardiomyocytes with 25 *μ*mol/L TMG, an OGA-specific inhibitor. Western blot showed that TMG treatment indeed significantly increased the level of O-GlcNAcyation in cardiomyocytes (Figure 4(a)). Consequently, the LC3-II level in cardiomyocytes was also increased significantly by TMG treatment, both at the basal level and with BafA (Figure 4(a)). Also, the pATG16L1 level was increased in cardiomyocytes treated with TMG (Figure 4(b)). Collectively, these data indicated that elevated O-GlcNAcylation in cardiomyocytes promotes autophagy.

### 3.5. ULK1 Is O-GlcNAcylated in Cardiomyocytes

**Figure 5:**
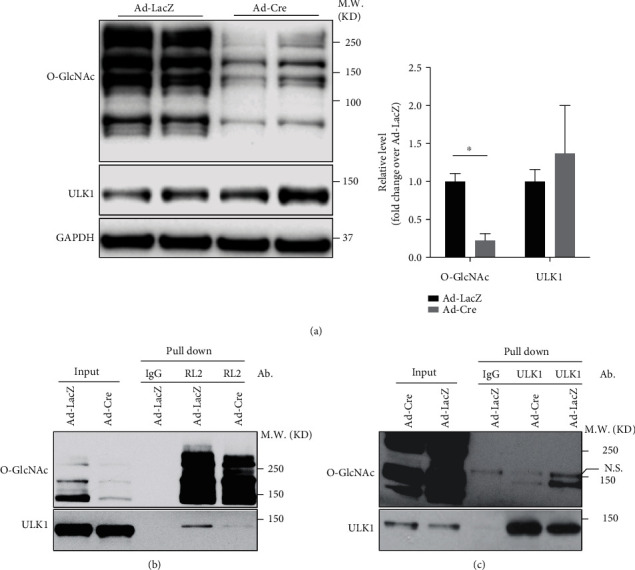
ULK1 is O-GlcNAcylated in cardiomyocytes. (a) Left panel, western blot analysis of Ad-LacZ- and Ad-Cre-infected *Ogt*^f/f^ and/or *Ogt*^f/y^ neonatal cardiomyocytes, using antibodies against O-GlcNAc and ULK1. GAPDH was detected as a loading control. Right panel, quantification of western blot results in the left panel, *n* = 4 for each group. ^∗^Significantly different; M.W.: molecular weight. (b) Immunoprecipitation of Ad-LacZ- and Ad-Cre-infected *Ogt*^f/f^ and/or *Ogt*^f/y^ neonatal cardiomyocytes using antibody against O-GLcNAc (RL2), followed by western blot using antibodies against O-GlcNAc (RL2) and ULK1. Ab.: antibody; M.W.: molecular weight. (c) Immunoprecipitation of Ad-LacZ- and Ad-Cre-infected *Ogt*^f/f^ and/or *Ogt*^f/y^ neonatal cardiomyocytes using antibody against ULK1, followed by western blot using antibodies against O-GlcNAc (RL2) and ULK1. Ab.: antibody; M.W.: molecular weight. N.S.: nonspecific band.

ULK1 is a key factor in controlling the initiation of autophagy [12]. Both *in vivo* and *in vitro* data strongly suggested that ULK1 is required for autophagy in cardiomyocytes [29]. In addition, ULK1 is also responsible for the phosphorylation of ATG16L1 [28]. Hence, we checked whether ULK1 was decreased in OGT deleted cardiomyocytes. Surprisingly, ULK1 showed an even higher protein level in OGT knockout cardiomyocytes (Figure 5(a)), excluding the contribution of decreased ULK1 to OGT deletion-induced autophagy attenuation. A recent report has shown that O-GlcNAcylation of ULK1 is required for ULK1-mediated autophagy in liver cells [19], which prompted us to examine whether ULK1 was O-GlcNAcylated in cardiomyocytes. Immunoprecipitation showed that ULK-1 was O-GlcNAcylated in cardiomyocytes. And the level of ULK1 O-GlcNAcylation was dramatically decreased in OGT knockout cardiomyocytes (Figures 5(b) and 5(c)). These data suggested that OGT control the initiation of autophagy in cardiomyocytes through the regulation of ULK1 O-GlcNAcylation.

## 4. Discussion

Previous studies have showed that cardiomyocyte OGT or O-GlcNAcylation was essential for cardiac function at both the basal level and under stress such as ischemia [6, 7], and the loss of OGT promoted cardiomyocyte's apoptosis [6, 7]. Interestingly, upregulation of autophagy during heart ischemia and reperfusion has also been shown to be cardiac-protective and prevented apoptosis of cardiomyocytes [30–32]. Therefore, we anticipated that the protective effect of O-GlcNAcylation in cardiomyocytes is mediated, at least partially, via the promoting of autophagy especially under stress. Indeed, our data from both *in vivo* and *in vitro* models clearly demonstrated that the loss of OGT in cardiomyocytes attenuated autophagy.

Interestingly, we found that the loss of O-GlcNAcylation in the heart decreased autophagy only when mice were subjected to starvation, while the loss of O-GlcNAcylation in isolated neonatal cardiomyocytes attenuated autophagy under both nutrient and starved conditions. One possible explanation is that during the process of isolation, the isolated cardiomyocytes might already had gone through stress, which increased the basic requirement of autophagy. Another explanation is that underdeveloped neonatal cardiomyocytes and mature adult cardiomcyotes might behave differently in the extent of autophagy regulations.

Of note, our results were different from the previous studies which showed that elevated O-GlcNAcylation blunted autophagy in the heart and cardiomyocytes [20, 21]. In those studies, STZ was used to elevate O-GlcNAcylation because STZ can induce hyperglycemia by damaging *β* cells. Consequently, STZ induces altered O-GlcNAcylation globally instead of just in cardiomyocytes, preventing the effects of O-GlcNAcylation in cardiomyocytes on autophagy to be elucidated. Also, STZ treatment in animals has numerous side effects [22], which further compromise the relationship between O-GlcNAcylation and autophagy inside cardiomyocytes. Our results from the OGT cardiomyocyte-specific knockout mouse model demonstrated that O-GlcNAcylation promotes autophagy in cardiomyocyte cell autonomously.

We also showed that O-GlcNAcylation is required for the early stages of autophagy because LC3-II level was decreased even with BafA treatment. Additionally, pATG16L1 level, a novel marker of newly formed autophagosome [28], was also decreased when OGT was knocked out in cardiomyocytes. Consistently, ULK1, a key regulator of autophagy at the early stage, was shown to be O-GlcNAcylated in cardiomyocytes, and O-GlcNAcylation of ULK1 was diminished when OGT was knocked out. In line with our findings, ULK1 cardiomyocyte-specific knockout mice show autophagy defects under stress [29]. Considering that ULK1 is a critical regulator of autophagy initiation and its O-GlcNAcylation is required for the induction of autophagy in other cell types [19, 33], we anticipate that OGT promotes autophagy through regulating ULK1 activity by O-GlcNAcylation in cardiomyocytes. Future studies are needed for confirmation.

In conclusion, using a cardiomyocyte-specific genetic deletion mouse model, for the first time, we demonstrated that O-GlcNAcylation is required for autophagy in cardiomyocytes especially under stress conditions. We also found that O-GlcNAcylation promotes initiation of autophagy probably through the regulation of ULK1 activity in cardiomyocytes. Our findings provide a better understanding of heart dysfunction and should be considered for their potentials in the prevention and treatment of heart failure.

## Data Availability

All data used to support the findings of this study are available from the corresponding author upon request.
